# @choo: Tracking Pollen and Hayfever in the UK Using Social Media

**DOI:** 10.3390/s18124434

**Published:** 2018-12-14

**Authors:** Sophie Cowie, Rudy Arthur, Hywel T. P. Williams

**Affiliations:** 1 Computer Science, College of Engineering, Mathematics and Physical Sciences, University of Exeter, Exeter EX4 4QE, UK; sophie_cowie@hotmail.com (S.C.); rudy.d.arthur@gmail.com (R.A.); 2 The Alan Turing Institute, 96 Euston Road, London NW1 2DB, UK

**Keywords:** hayfever, social media, pollen, social sensing, crowdsourcing

## Abstract

Allergic rhinitis (hayfever) affects a large proportion of the population in the United Kingdom. Although relatively easily treated with medication, symptoms nonetheless have a substantial adverse effect on wellbeing during the summer pollen season. Provision of accurate pollen forecasts can help sufferers to manage their condition and minimise adverse effects. Current pollen forecasts in the UK are based on a sparse network of pollen monitoring stations. Here, we explore the use of “social sensing” (analysis of unsolicited social media content) as an alternative source of pollen and hayfever observations. We use data from the Twitter platform to generate a dynamic spatial map of pollen levels based on user reports of hayfever symptoms. We show that social sensing alone creates a spatiotemporal pollen measurement with remarkable similarity to measurements taken from the established physical pollen monitoring network. This demonstrates that social sensing of pollen can be accurate, relative to current methods, and suggests a variety of future applications of this method to help hayfever sufferers manage their condition.

## 1. Introduction

Allergic rhinitis (hayfever) is an unpleasant disease which affects one in five 16–44 year olds, and up to 35% of 13–14 year olds in the UK [1,2]. Symptoms such as sneezing, runny nose, wheezing and itchy eyes can impact on work productivity and quality of life [3]. Health effects can be severe, especially if combined with other conditions such as asthma which can lead to complications and sometimes hospitalisation. Over 80% of asthmatics have allergic rhinitis and 10–40% of patients with allergic rhinitis have asthma [4].

Currently, forecasts of pollen levels in England and Wales are based on a sparse network of 14 observation stations [5], which collect pollen at two-hourly intervals and are averaged daily, combined with weather forecast variables, pre-season weather information, local vegetation information and biological factors. Recent work by McInnes et al. [6] has improved understanding of the spatial distribution of 12 types of allergenic vegetation by producing high resolution maps based on observed and modelled data sets. However, the overall system of pollen forecasting and monitoring could be improved by an alternative source of data. Some recent studies have considered novel data sources for this purpose, for example, remote sensing by satellite [7] and smartphone applications [8]. In this study, we explore the utility of ‘social sensing’, systematic analysis of social media data, to track pollen and hayfever symptoms. If reliable observations can be generated from this novel data source, it offers several advantages. Another data source allows triangulation and cross-validation of existing (sparse) observations, potentially leading to forecast model improvements by better validation. Social media users are widely dispersed, potentially allowing greater spatial coverage and resolution of observations, compared to the 14 observing stations in the existing physical sensor network. Finally, since social media data about hayfever is likely to be generated primarily by sufferers, social sensing provides direct observations of the human impact of pollen and hayfever, something that must otherwise be inferred from pollen counts.

Social sensing, beginning with the work of Sakaki et al. [9] on earthquakes, has proven successful at identifying and tracking extreme weather and other disruptive natural phenomena, e.g., wildfires [10], air pollution [11], and floods [12]. We distinguish social sensing from solicited crowd-sourcing, where users are recruited to participate and generally report their observations in a structured or semi-structured manner. Many citizen science and crowdsourcing efforts rely on solicited observations, e.g., the UK Met Office ‘Weather Observation Website’ [13] or the UKSnowMap [14] that uses a particular hashtag (#uksnow) to allow users to report snowfall observations via Twitter. The benefit of these efforts is that mostly reliable data, from dedicated volunteers, is collected through a structured interface. However, the (typically low) volume of data collected can limit their usefulness.

Social sensing is a form of unsolicited crowdsourcing which collects observations from social media. These may be relevant and useful to forecasters but are not produced for this purpose and unlikely to follow a consistent reporting structure. A single observation (e.g., “My hayfever is really bad today!”) tied to a reasonably precise location may not alone allow precise models to be built, but by aggregating thousands of such observations over significant time periods, we aim here to show that accurate temporal and spatial distributions can be obtained for pollen and associated hayfever symptoms. The main technical challenge in social sensing is to extract high-quality observational data—relevant information tied to precise locations—from large numbers of unstructured, noisy, and possibly inaccurate user posts and their associated metadata.

The natural events for which social sensing has been successful tend to be extreme, episodic, as well as temporally and spatially bounded. Earthquakes and floods are prime examples. Social media users in a small area in a short time typically generate a low background number of tweets on a particular topic, but an extreme or unexpected event can generate a signal large enough to detect above the background of daily chatter.

Similar in approach to the use of social sensing to track environmental hazards is the body of work that uses social media to track health [15]. This field has typically been concerned with tracking epidemics (e.g., [16]) or correlating user sentiment with health (e.g., [17]). Hayfever is at the intersection of these two areas, in that it is an environmentally triggered health condition which people discuss on social media, while also being spatio-temporally bounded. The UK peak pollen season is June (early Summer) and its seasonal nature suggests it will generate a reasonable volume of social media discussion when it occurs. Since hayfever symptoms require sufferers to be near an allergenic pollen source, the social media observations they may produce are likely to be co-located with pollen.

In this study, we will explore the feasibility and accuracy of social sensing of hayfever and pollen. Primarily, we will compare hayfever- and pollen-related Twitter activity, suitably filtered and normalised, to observed pollen counts in the regions of the existing monitoring stations. Secondary comparisons will look at which kinds of pollen produce the greatest social response, as well as correlations with other sources of web data. The final part of the analysis will examine the performance of simple linear regression models that predict pollen count as a function of social sensing observations. If such models are shown to be effective, they might be useful for providing estimated pollen counts in areas where there is no monitoring station or in the case of monitoring station failure.

The manuscript is structured as follows: Section 2 describes the datasets and methods used; Section 3 describes the empirical results and gives some interpretation; Section 4 tests the performance of simple predictive models; and Section 5 summarises the main conclusions from the work.

## 2. Data Collection and Methods

### 2.1. Social Media Data

Twitter data collection methods follow those reported in [12]. The keywords *hayfever* and *pollen* were used to collect tweets over the time period December 2016–November 2017 via the Twitter Streaming API. The tweets are returned as JSON objects, including the tweet text and metadata associated with the tweet, such as timestamp, user profile details and location (if provided by the user).

Overall, the tweet collection contained 771,707 tweets. Although other social sensing studies record more tweets (e.g., [12] sees single days with the word ‘flood’ mentioned in over one hundred thousand tweets), the volumes reported here are a reflection of both the severity and ubiquity of hayfever. Hayfever symptoms are not as notable as large flood events. Hayfever is also relatively common and therefore perhaps less worthy of mention on social media. However, the volumes of data obtained here are sufficient to allow robust analysis, so long as care is taken over the spatial areas taken as sampling units (see below).

The initial set is filtered to only include tweets authored by users whose profile indicates a UK timezone. Others are assumed to refer to conditions elsewhere than the UK and are irrelevant for this study. As of May 2018, in order to comply with General Data Protection Regulation (GDPR) requirements, Twitter has removed the timezone field from tweet meta-data. Other methods exist (e.g., [12]) to identify user location. Alternatively, the Twitter API can return data from a given geographical bounding box.) This leaves 53,741 tweets. The next stages of filtering are:

1. **Removing tweets from automated ‘bot’ accounts.** Tweets that are produced automatically do not contain any useful information for detecting hayfever complaints. We first identified accounts that produced the largest volume of tweets, since bots typically create orders of magnitude more tweets than regular users. Initially, we chose a filter where an account was deemed a ‘bot’ if it contributed more than 1% of the total volume of tweets in the collection. An additional manual review of the top 100 most frequently tweeting accounts identified additional automated accounts, which we also removed. Most of the bots removed were marketing accounts focused on health and lifestyle products.
2. **Removing retweets.** We wish to focus on original information, posted by the person who is themself suffering from hayfever. As retweets involve sharing someone else’s tweet, potentially at a different location, we are less confident that the information in a retweet is relevant. To remove this uncertainty, we filter out retweets.
3. **Applying machine learning to remove irrelevant tweets.** This step removes tweets which are articles, business advertising or do not represent a hayfever symptoms, for example (fictional) ‘Hurray, no hayfever today!’. We manually curated a set of 1015 tweets, classifying each tweet as relevant or not, and used them as training data for a Multinomial Naive Bayes [18] classifier. These tweets were randomly chosen and manually classified as relevant or irrelevant. We used 1-g and 2-g extracted from the tweet text as the features on which to classify whole tweets; testing showed that adding higher n-grams provided no additional benefit. This relevance filtering step also helps to remove any automated tweets which were not captured in Stage 1. Using 25% of the data as a validation set the classifier correctly identified the relevance of 90% of the tweets. A 6-fold cross validation gave an $F_{1}$ score of 0.94 and the confusion matrix below:
  $$\left(\begin{array}{cc} & Predicted\\ \mathrm{Actual} & \begin{array}{ccc} & I & R\\ I & 121 & 80\\ R & 16 & 798 \end{array} \end{array}\right).$$
  This confusion matrix implies a slight tendency to over-classify tweets as relevant. This filter is accurate and sufficient for our purposes, removing most of the irrelevant tweets. In future work, it could be improved with more training data, particularly more examples of irrelevant tweets.

These filters together result in 64% of our original UK-timezone tweets being discarded, leaving 19,316 tweets with useful information.

Location information is important for mapping the spatial distribution of hayfever tweets. However, only 0.3% of tweets in this dataset were geo-tagged by the user, i.e., the user chose to attach a precise GPS tag to that particular tweet. Where geo-tagged information is available we used it, but otherwise we used the location from the user’s profile page. We primarily used the online gazetteer *geonames* [19]. We attempted to match all n-grams from the user location field against entries in *geonames*. We found that 52% of Tweets had some form of useable location information i.e., city, town or county names. Using only the user location is not ideal as it is unlikely that a user will only tweet from the location on their profile. Unlike in flood detection, where location information is often included in the text to identify flooded areas [12], this is not so common for hayfever complaints as the message is typically more about how the tweet author is feeling, rather than where they are experiencing the symptoms. Nevertheless, there are enough tweets with geographic information to allow robust statements about local intensities and seasonal trends, when aggregated over sufficiently large areas (see below).

### 2.2. Pollen Count Data

UK pollen data for the same period was collected from the network of pollen monitoring stations [5] run by the UK Met Office in collaboration with the National Pollen and Aerobiology Research Unit (NPARU) [20] based at the University of Worcester. The locations of the 11 stations used in this study are shown in Figure 3.

The data are collected using a seven-day volumetric spore trap made by Burkard, which operates in a similar way to the original trap described in [21], but with improved trapping efficiency for particles in the range 1–10 μm diameter. The UK network of pollen observation stations records the pollen count for the three most allergenic types of vegetation; grass, tree and weed. Data are supplied for twelve different pollen types, split into eight different tree species, three weed and one grass as a daily average pollen count for each type. The relative contribution of tree, grass and weed pollen to the UK allergy burden is unclear. Despite grass pollen measurements being consistently lower than trees, grass pollen allergy has been found to be more common in the UK [22].

### 2.3. Other Data

We used Google Trends [23] to get data on how frequently web users conducted searches containing the words *hayfever* or *pollen*. The output from Google Trends is a tabulated file (downloaded manually) which contained here the percentage of searches relative to the peak number of searches for each week over the 12-month period for the UK only.

The Met Office provided web traffic data (number of visits) for their pollen pages, accumulated weekly for the period 1 February 2017 to 31 October 2017. We normalised these data by the peak value over the time period so they were in the same format as Google Trends data.

## 3. Empirical Results and Discussion

In this section, we first consider empirical pollen count data for the 11 monitoring stations in England and Wales, and compare this data to tweets and other online data aggregated over the same area. Then, we compare tweets in the area local to each station separately. In the following section, we explore whether tweets could be used in predictive models to improve the estimate of pollen count in areas remote to stations.

### 3.1. Pollen Count Seasonality

**Figure 1:**
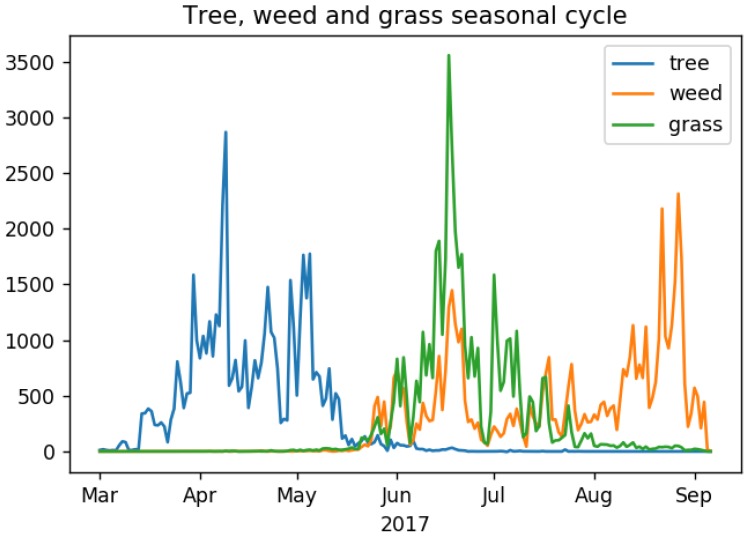
Time-series of total UK daily pollen counts (*y*-axis), grouped by vegetation type. Plot shows: tree pollen (blue), weed pollen (orange), grass pollen (green).

We observe three very different seasons for tree, grass and weed pollen in the pollen observation data (Figure 1). Tree pollen count starts to rise in March, peaks in early April, then decreases through May. Grass and weed pollen count increase dramatically in June, with weed pollen having a second peak later in August (Figure 1). These seasons are in agreement with information on the ‘Pollen Calendar’ produced by the National Pollen and Aerobiology Research Unit [24] and other studies of pollen allergies [22].

### 3.2. Comparison of Pollen Counts with Social Data Sources

**Figure 2:**
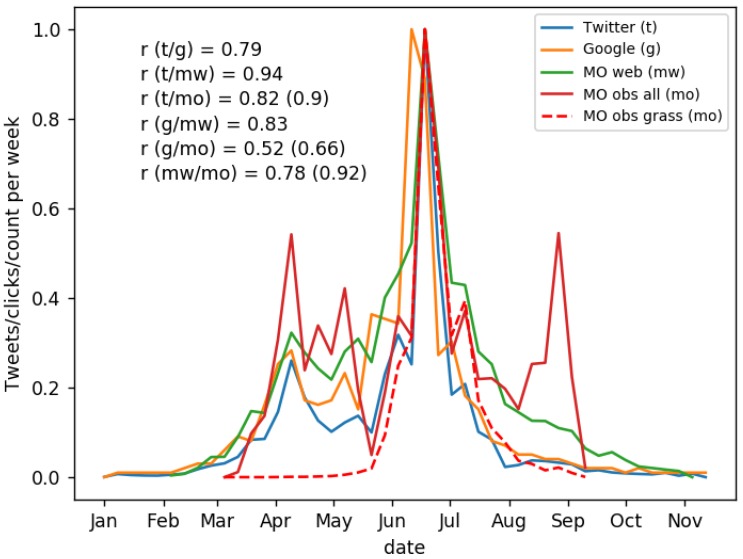
Time-series of various data sources for monitoring pollen and hayfever, aggregated weekly and normalised by their maximum value over the time period. Plot shows: Normalised Twitter activity (*t*, blue), Google Trends web search activity (*g*, orange), Met Office web traffic (*mw*, green), counts for all pollen (*mo*, solid red) and grass pollen (*mo*, dashed red). Correlations, r, are given for each data source pair; pollen count correlations are for all pollen, with correlations for grass pollen only given in brackets.

All data were aggregated into weekly observations because of sparsity in the observations, especially during periods with low pollen count. Weekly aggregated Twitter activity correlates well with other data sources such as Google searches (r=0.79), Met Office web traffic (r=0.94) and Met Office pollen observations (r=0.82), as shown in Figure 2. Using grass pollen observations only increases the correlation (r=0.9).

Figure 2 also shows a stronger Twitter response to grass pollen than to tree pollen, and very little Twitter response to weed pollen. Twitter activity has closer agreement to all-pollen counts from Met Office observations in the June peak, and to a lesser extent in the April peak, corresponding to grass and tree pollen respectively, with little agreement for the August–September weed pollen peak. These observations suggest that most hayfever sufferers are allergic to grass pollen, fewer to tree pollen, and very few to weed pollen. Similar patterns are seen for Google Trends and Met Office web traffic, which also improve when only grass-pollen observations are used (*r*-values in brackets in Figure 2).

These findings help characterise the observations possible with social sensing. Social reporting of hayfever, as might be expected, focuses on allergies and symptoms, rather than the pollen itself. Our results confirm the stronger allergenic effect of grass compared to tree or weed pollen.

### 3.3. Local Comparison of Twitter Data and Pollen Counts

**Figure 3:**
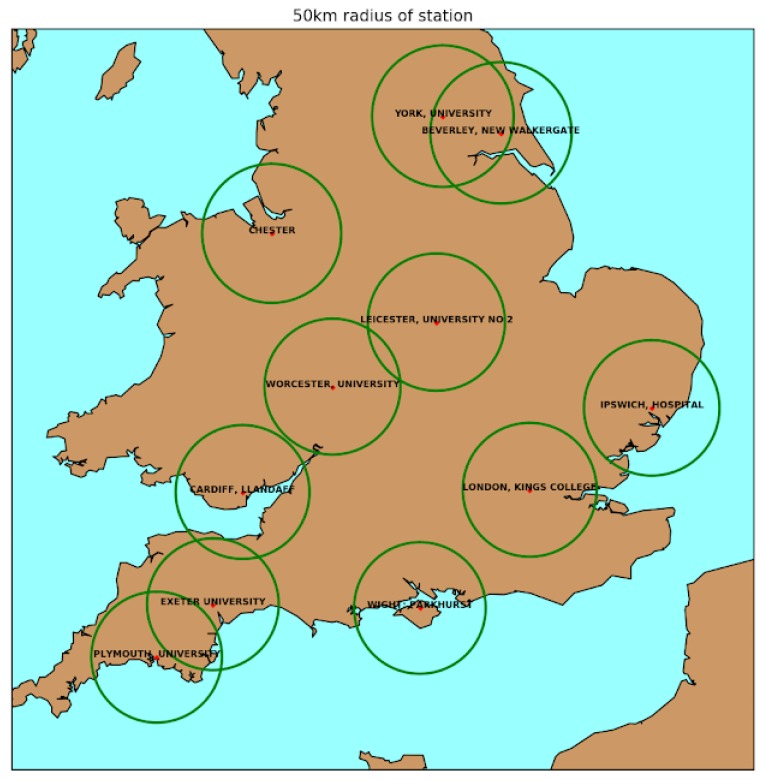
Pollen monitoring station locations (red dots), with green circles indicating the 50 km radius around each station.

After demonstrating that pollen counts aggregated nationally are strongly correlated with web data, we also investigated the relationship between tweets and pollen counts at a local level. The locations of pollen monitoring stations are shown in Figure 3. A time series of tweets from users located within a 50 km radius of each station was aggregated weekly and correlated with a time series of weekly averaged pollen counts.

Most inferred-location tweets were located to a town or city located completely inside or outside one of the 50 km radii. Border cases, where the tweet location was inferred to a polygon which intersected, but was not contained in, one of our study areas were decided based on the position of the centroid of the inferred tweet polygon. For cases where the 50 km radii of two stations intersect (e.g., Exeter and Plymouth study areas), we have some double counting of tweets, which will artificially inflate the correlation between those two stations. However, in practice, there were very few total tweets in these intersections; for example, the Exeter/Plymouth overlap encompasses the mostly unpopulated Dartmoor area, so we expect the effect to be small. Furthermore, our main method is correlating tweet volumes with pollen counts at a single station, where artefacts from double counting are not as much of an issue. The 50 km radius was chosen as the smallest distance where we had a sufficient volume of tweets around each station to compute reliable correlations. These correlations were computed for all-pollen counts and grass-pollen counts separately, shown in Table 1.

Local correlations with all-pollen counts are strong, while again correlations are typically stronger for grass pollen only (Table 1). The Leicester station has an unexplained two-week gap in the observational data, occurring at a time that measurements from the tweet time series, and other monitoring stations, suggests is peak pollen season. This explains the low correlation with low significance at that station. Also worth noting is the high variability in the number of tweets observed close to each station, explained by the varying population densities in the different areas.

## 4. Prediction of Pollen Counts Using Models Including Social Sensing Data

Given that the number of pollen- and hayfever-related tweets is strongly correlated with the observed pollen count at each station, we can try to use the number of tweets to help predict pollen counts in areas without pollen observation stations. In the following analysis, we investigate whether adding tweet counts improves the performance of simple models that predict pollen levels for a given location based on levels at nearby areas. For comparison, we also consider a tweets-only model. All of the following analysis focuses on grass pollen only.

### 4.1. Model Construction

We formulate and test a series of linear regression models using different combinations of Twitter and pollen data, fitting parameters using least-squares optimisation. We test the power of a model using ‘leave one out’ validation. If we let *x* be the position of pollen station *j*, then we can predict the pollen count P(x) at that location using the other stations and compare to the observed count at *j*. The results shown in Table 2 use weekly aggregate pollen counts and compare across our whole observation period.

#### 4.1.1. Model 1—Nearest Pollen Station

The simplest model for predicting the pollen count at a point *x* would be to use the pollen count recorded at the station nearest to *x*. Thus:

(1) $$P_{1}\left(x\right)=a_{1}p_{i}+c_{1},$$

where $p_{i}$ is the pollen count recorded at station *i* (the nearest station to point *x*), $a_{1}$ is the slope and $c_{1}$ the intercept of the regression line, obtained by by fitting the model to observations. We will call this Model 1.

#### 4.1.2. Model 2—Weighted Average of Pollen Stations

A slightly better approach is to use a weighted average of observations at all stations (except the target station), where the weights are inverse distances to the target location for which we want to make a prediction. Let:

(2) $$p^{\prime }\left(x\right)=\sum_{i}p_{i}w_{i}\left(x\right)/\sum_{i}w_{i}\left(x\right),$$

where $w_{i}\left(x\right)=\frac{1}{d(i,x)}$ and d(i,x) is the distance between each pollen station *i* and the point *x*. Then:

(3) $$P_{2}\left(x\right)=a_{2}p^{\prime }\left(x\right)+c_{2}.$$

$a_{2}$ is the slope and $c_{2}$ the intercept of the regression line, obtained by fitting the model to observations. This is Model 2.

#### 4.1.3. Model 3—Local Tweets

We can investigate how well tweets predict pollen counts locally by using a simple linear model:

(4) $$P_{3}\left(x\right)=a_{3}t\left(x\right)+c_{3},$$

where t(x) is the number of tweets within some sampling radius of the point *x* recorded during the week in question, and $a_{3}$ is the slope and $c_{3}$ the intercept of the regression line, obtained by by fitting the model to observations. Since our strongest local correlations were made with radii of 50 km, we only show results for this value. This is Model 3.

#### 4.1.4. Models 1T & 2T—Pollen and Tweets

The best model should combine all information sources. Adding tweets to the ‘Nearest Pollen Station’ (Model 1) formulation gives Model 1T:

(5) $$P_{1T}\left(x\right)=a_{1T}p_{i}+b_{1T}t\left(x\right)+c_{1T},$$

while adding tweets to the ‘Weighted Average of Pollen Stations’ (Model 2) formulation gives Model 2T:

(6) $$P_{2T}\left(x\right)=a_{2T}p^{\prime }\left(x\right)+b_{2T}t\left(x\right)+c_{2T},$$

where $a_{1T},\ldots ,c_{2T}$ are, as before, parameters found by fitting the models to data.

### 4.2. Model Performance

Performance of Models 1–3, as well as comparisons between $P_{1}\left(x\right)$ and $P_{1T}\left(x\right)$ and between $P_{2}\left(x\right)$ and $P_{2T}\left(x\right)$, are shown in Table 2. Model performance is reasonable, showing that with or without Twitter data, it is possible to make a good estimate of the pollen count at a location based on counts at nearby locations. Tweet data alone does not predict pollen as well as using pollen counts from other stations. Adding Twitter data slightly improves the fit quality and reduces the RMSE for Model 1 compared to Model 1T, with a smaller effect on Model 2T compared to Model 2. While Model 3 (tweets only) is the worst performing model, it still shows a strong correlation and reasonable performance demonstrating that predictive models could also be made from social sensing data alone if pollen counts were completely unavailable. In fact, upon further investigation, the low correlation observed with the Leicester station was a result of a collection ‘outage’ and reporting of out of date pollen counts for the time period in question. This already shows that this method has some practical value as a simple check on any potential anomalies in the reported pollen count.

## 5. Conclusions

Twitter is a rich source of information on the spatial and temporal distribution of pollen in the UK and its human impact. We demonstrated that the normalised volume of tweets correlates highly with the seasonality of pollen count and hayfever and that selecting grass pollen alone increases these correlations. This supports the widely claimed, but not widely proven, belief that people are more allergic to grass pollen than tree or weed pollen. This assertion is otherwise very difficult to test, beyond performing large scale and expensive epidemiological surveys, and suggests potential further applications for Twitter in measuring the temporal profile of common conditions like flu, asthma or seasonal depression.

The seasonal correlations between pollen counts and social sensing data are strong at both national and local scales. This implies that social sensing could provide estimates of pollen levels in areas remote from observing stations, provided there is a consistent social media presence there. One limitation of the approach demonstrated here is that the volume of data available was rather sparse. Using the Twitter Streaming API, the total volume of relevant content was sufficient to give robust estimates for the 50 km radius sampling areas tested, but might prove problematic for mapping at finer spatial or temporal resolutions. One possible improvement in this aspect would be to use the full Twitter data stream, rather than the limited sample that is publically available through their API; since this is only available commercially, we were not able to test this idea.

As a further test of this methodology, in preliminary work, we also attempted to perform similar analysis for related environment/health conditions, specifically asthma and air pollution. This exploration showed that there was very little relevant data available for these topics, certainly not enough to make social sensing effective. We speculate that the lack of data for asthma might be because people use a variety of terminology for this condition (e.g., ‘wheezy’, ‘breathless’, ‘tight’) and our keywords were inaccurate. Alternatively, it is equally possible that asthma sufferers do not discuss their condition in the same way as hayfever sufferers. Hayfever is seasonal, and, as such, it is noteworthy when it occurs. Asthma is a year-round chronic condition and perhaps less noteworthy as a result. The lack of data on air pollution was surprising given findings elsewhere, specifically Jiang et al. [11], who reported success with this method in Chinese cities. Our conclusion based on inspection of the content we found for the UK is that in the UK air pollution is mainly discussed on Twitter as a political concern; most tweets that referenced the topic were discussing legislation to control air pollution or public debate of the issue, rather than reporting personal experiences of air pollution as an environmental hazard. It is possible that our shallow initial explorations of social sensing for asthma and air pollution might have been more fruitful with a more sophisticated data collection strategy.

With more and better located social data we could further improve pollen predictions. Worth noting is that this analysis only uses Twitter, and adding other sources such as Facebook or Instagram may improve the results, especially as the users on these platforms may choose the share different types of information compared to Twitter. Furthermore, here we have only analysed the volume of social media activity, without analysing the content; it is possible that text analysis of content may provide additional information that could further improve predictive models. As it stands, if Twitter usage continues in the same manner, these models have a useful role in monitoring pollen levels. If Twitter were to change its platform, or some other reason were to alter the level of usage by hayfever sufferers, these models would need to be re-trained or abandoned.

The greatest value in tweets is the human impact information; people are communicating their actual experience with hayfever, and this is difficult information to get otherwise. This paper describes how to use social media to detect spatial and temporal trends in allergy symptoms, and how to combine simple count data to get better measurements. Better measurement of hayfever incidence and severity can aid healthcare planning and delivery, as well as enabling individual sufferers to manage their condition. Scientific understanding of pollen and hayfever can also benefit from alternative data sources, including for the improvement and validation of forecasts.

## Figures and Tables

**Table 1:** Pearson’s *r*, for the relationship between number of tweets within a 50 km radius of each pollen observation station and the pollen counts observed at each station, for all pollen types and grass pollen only. Text colour indicates *p*-value (Red: p≥0.05, Blue: 0.01≤p<0.05, Black: p<0.01).

	Correlation Between GRASS Pollen Counts and Tweets	Correlation Between ALL Pollen Counts and Tweets	No. of Tweets in 50 km Radius of Station
York	0.9	0.76	637
Beverley	0.88	0.88	271
Chester	0.87	0.69	617
Leicester	0.44	0.07	790
Worcester	0.83	0.83	814
Ipswich	0.66	0.47	164
London	0.59	0.59	2325
Cardiff	0.88	0.55	544
Plymouth	0.8	0.53	217
Exeter	0.68	0.52	235
Wight	0.84	0.82	405
MEAN	0.76	0.61	638.1

**Table 2:** Model performance with/without Twitter data. Table shows $R^{2}$ (coefficient of determination) and root mean squared error (RMSE) for Models 1, 2, 3, 1T and 2T. All scores are averaged from leave-one-out validation tests across all 11 pollen monitoring stations.

	$R^{2}$	RMSE
Model 1	0.810	0.115
Model 2	0.847	0.099
Model 3	0.745	0.188
Model 1T	0.858	0.100
Model 2T	0.858	0.096
